# Supplementary material for: Mapping protein selectivity landscapes using multi-target selective screening and next-generation sequencing of combinatorial libraries
Source: Nat Commun. 2018 Sep 26;9:3935. doi: 10.1038/s41467-018-06403-x (PMC6158287; doi:10.1038/s41467-018-06403-x)
Supplement: Supplementary file 1 — Supplementary Information [file 41467_2018_6403_MOESM1_ESM.pdf]

Mapping protein selectivity landscapes using multi-target selective screening and next-generation sequencing of combinatorial libraries

Naftaly et al.

Si Naftaly and Itay Cohen contributed equally to this work

SUPPLEMENTARY INFORMATION

Supplementary Methods

Generating a protein library based on APPI-3M.

The library was constructed based on our previously published $APPI_{M17G/I18F/F34V}$ sequence (APPI-3M)[1] along with a peptide linker (NH_3^+-APPI-LPDKPLAFQDPS-COO^-) using codon optimization for *Saccharomyces cerevisiae* and *P. pastoris* usage. The library was generated by PCR-assembly using the following ten overlapping oligonucleotides (5'-3'):

(1) GGTGGTTCTGGTGGTGGTGGTTCTGGTGGTGGTGGTTCTGCTAGCGAAGTTTGTTCTGAACAAGCTG

(2) GAAGTTTGTTCTGAACAAGCTGAA**NNSGGTCCATGTAGAGCTGGTTTT**TCTAGATGGTATTTCGATGTTACTG

(3) GAAGTTTGTTCTGAACAAGCTGAA**ACTNNSCCATGTAGAGCTGGTTTT**TCTAGATGGTATTTCGATGTTACTG

(4) GAAGTTTGTTCTGAACAAGCTGAA**ACTGGTNNSTGTAGAGCTGGTTTT**TCTAGATGGTATTTCGATGTTACTG

(5) GAAGTTTGTTCTGAACAAGCTGAA**ACTGGTCCATGTNNSGCTGGTTTT**TCTAGATGGTATTTCGATGTTACTG

(6) GAAGTTTGTTCTGAACAAGCTGAA**ACTGGTCCATGTAGANNSGGTTTT**TCTAGATGGTATTTCGATGTTACTG

(7) GAAGTTTGTTCTGAACAAGCTGAA**ACTGGTCCATGTAGAGCTNNSTTT**TCTAGATGGTATTTCGATGTTACTG

(8) GAAGTTTGTTCTGAACAAGCTGAA**ACTGGTCCATGTAGAGCTGGTNNS**TCTAGATGGTATTTCGATGTTACTG

(9) GATGGTATTTCGATGTTACTGAAGGTAAATGTGCTCCATTCGTCTATGGTGGTTGTGGTGGTAATAGAAATAATT TCGATACTGAAGAATATTGTATGGCTGTTTGTGGTTCTGCTATTGGATCCTTGCCAGATAAACCATTGGCTTTCC

(10) GAGCTATTACAAGTCCTCTTCAGAAATAAGCTTTTGTTCAGATGGATCTTGGAAAGCCAATGGTTTATC

The synthetic library was assembled by a set of three PCRs using low-fidelity Taq polymerase, as follows: in the first reaction, oligos number 2–9 were incorporated using error-prone PCR to generate both site-specific saturation randomization of the binding loop residues (residues 11–18, except residue 14, using NNS codons, where N = A/C/G/T and S= C/G) and an arbitrary randomization throughout the whole sequence to include their neighboring and other scaffold residues. The error-prone PCR reaction was optimized to 15× PCR doublings using a low-fidelity Taq polymerase, 1% nucleotide analogues, and 2 mM $MgCl_2$. Next, the assembled fragment was amplified by PCR using 5'-GAAGTTTGTTCTGAACAAGCTG-3' and 5'-GGAAAGCCAATGGTTTATC-3' primers, followed by a final PCR reaction using oligos number 1 and 10.

The final PCR-assembled fragment was gel-purified and cloned into a pCTCON vector via transformation by electroporation of *EBY10*0 yeast cells and homologous recombination with the linearized vector (digested with *Nhe*I and *Bam*HI). Random and site-specific mutagenesis (error-prone and NNS) in the APPI sequence generated a library

of about 3.5×10^6 variants, as estimated by dilution plating on selective SDCAA plates (15% agar, 2% dextrose, 1.47% sodium citrate, 0.429% citric acid monohydrate, 0.67% yeast nitrogen base, 0.5% casamino acids). After a high-throughput sequencing (Hylabs, Rehovot, Israel), the average amino acid mutation rate in the library was determined to be 0–2 mutations per 56 amino acids of the APPI sequence, as estimated from 10^6 sequences.

Library preparation for high-throughput sequencing. A YSD vector (pCTCON) containing the APPI gene was isolated from the naïve and sorted libraries by using the USB PrepEase Yeast Plasmid Isolation Kit (Affymetrix, Santa Clara, CA) according to the manufacturer's protocol. Using this kit, ~65 µg of pCTCON were isolated from each yeast library. Subsequently, 1 ng of the isolated plasmid was used for PCR amplification. The reaction included the following procedures and reagents: annealing temperature of 60 °C, 2% DNA template, 5% FWD primer (10 µM), 5% REV primer (10 µM), 20% HF buffer, 2% dNTPs, and 1% phusion HF polymerase in double-deionized water (DDW). The PCR conditions were as follows: denaturation for 10 s at 98 °C; annealing for 30 s at 60 °C; extension for 15 s at 72 °C; 30 cycles. The first PCR product was sent to the NGS laboratory of Hy Laboratories (Hylabs, Rehovot, Israel) for a secondary PCR of eight cycles, using the Fluidigm Access Array primers to add the adaptors and barcodes. Then, the DNA library samples were purified with AmpureXP beads (Beckman Coulter, Brea, CA) and the concentrations of the samples were determined in a Qubit by using the DNA high sensitivity assay. The samples were pooled and then ran on a TapeStation (Agilent, Santa Clara, CA) to verify the size of the PCR product. As a final quality test, the pools were subjected to qRT-PCR to determine the concentration of the DNA that can be sequenced. The pools were then loaded for sequencing on an Illumina Miseq, using the 500v2 kit.

Protein expression and purification. The selected APPI-3M variants were expressed in the *Pichia pastoris* strain *GS115*. To this end, EcoRI and AVRII restriction sites (New England Biolabs, Ipswich, MA) were added to the DNA sequences of each variant through a PCR reaction. Then, the obtained DNA sequences were digested using EcoRI and AVRII and ligated to a pPIC9K plasmid, which was digested with the same enzymes. Consequently, a His tag was added to the C-termini of the APPI-3M variants. The ligated plasmids were confirmed by transformation into *E. coli* and the sequencing of the

extracted DNA [DNA Microarray and Sequencing Unit (DMSU), the National Institute of Biotechnology in the Negev (NIBN), BGU]. Finally, the plasmids were linearized by a SacI restriction enzyme (New England Biolabs) and transformed into *P. pastoris* by electroporation. Thus, the sequenced variants were inserted into the alcohol oxidase (*AOX1*) locus of *P. pastoris*. The transformed cells were grown for two days at 30 °C on RDB plates [18.6% sorbitol, 2% dextrose, 1.34% yeast nitrogen base, 4×10^{-5} % biotin, and L-glutamic acid, L-methionine, L-lysine, L-leucine, and L-isoleucine (0.005% each)], selected, and, for further selection of multiple copies, plated on YPD-G418 plates (Geneticin, 4 mg ml^{-1}, Invitrogen, Carlsbad, CA) for 2 days at 30 °C. The genomic DNA of the selected cells was sequenced (DMSU, NIBN, BGU) to verify the insertion of the correct APPI variant. After the verification, the selected cells were grown overnight at 30 °C with a 300-rpm shaking in a 5-ml BMGY medium (2% peptone, 1% yeast extract, 0.23% $K_2H(PO_4)$, 1.1812% $KH_2(PO_4)$, 1.34% yeast nitrogen base, 4×10^{-5} % biotin, 1% glycerol). Expression was then induced by growing the cells for 3 days in a 5-ml BMMY medium (same as BMGY, but with 0.5% methanol instead of glycerol), while 0.5% methanol was added daily. The cells were suspended by centrifugation and the overexpression of the APPI-3M variants was tested by their ability to inhibit the catalytic activity of bovine trypsin. The cells with the highest overexpression of each variant were inoculated into 50 ml of BMGY medium, grown overnight at 30 °C with a 300-rpm shaking (until an $O.D_{600}$ of 10 was reached) and then grown in 500 ml of BMMY medium for 5 days, with 2% methanol added daily. After 5 days, the cells were removed by two rounds of centrifugation, and the supernatant with the expressed APPI-3M variants was filtered with a 0.22 µm Steritop bottle-top filter (Millipore). The pH of the filtered supernatant was adjusted to 8.0, and 300 mM of NaCl and 5 ml of 1 M imidazole (final concentration of 10 mM) were added and incubated for 1 h at 4 °C. Then, the APPI-3M variants were purified by using a nickel-immobilized metal affinity chromatography (IMAC) on a HisTrap 5-ml column (GE Healthcare, Piscataway, NY). The washing buffer contained 20 mM sodium phosphate, 0.5 M NaCl, and 10 mM imidazole (pH 8.0) and the elution was conducted by using an elution buffer (similar to the washing buffer, but with the addition of 0.5 M imidazole) in an ÄKTA pure instrument (GE Healthcare, Buckinghamshire, England). The eluted variants were concentrated and the buffer was replaced by a binding buffer (100 mM Tris, pH = 8.0, 1 mM $CaCl_2$, 1% BSA) by using Vivaspin with a 3-kDa cutoff (Vivaproducts, MA, USA). Subsequently, the variants were

purified with a Superdex 75 16/600 size-exclusion column (GE Healthcare) in an ÄKTA pure instrument. The molecular mass of the pure APPI variants was validated by mass spectrometry (MALDI-TOF REFLEX-IV, Bruker, Germany) at the Ilse Katz Institute for Nanoscale Science and Technology, BGU.

The yield of each purification was 0.4–0.9 mg per 500 ml of yeast culture, as measured with a NanoDrop spectrophotometer (Thermo Scientific, Waltham, MA), based on absorbance at 280 nm with an extinction coefficient 13,325 $M^{-1}cm^{-1}$, and with an inhibitor titration by activity assay, using bovine trypsin according to an established protocol[2]: Bovine trypsin was mixed with a range of substoichiometric concentrations of APPI and then assayed for residual activity with the substrate Nα-benzoyl-L-arginine 4-nitroanilide (Sigma, Poole, UK); a plot of residual enzyme activity versus APPI concentration allowed extrapolation to the stoichiometric equivalence point.

All trypsins were expressed in *E. coli* and purified as previously described[1,3]: Recombinant human anionic trypsinogen, human cationic trypsinogen, human mesotrypsinogen, and the catalytically inactive S195A mutants of these pro-enzymes were expressed in *E. coli*. Inclusion bodies were isolated and washed with 2 M urea, 2% Triton X-100, 50 mM Tris pH 8.0, 10 mM EDTA, and 5 mM DTT, and then trypsinogens were solubilized in 4 M guanidine-HCl, 0.1 M Tris pH 8.0 and 30 mM DTT. Trypsinogens were diluted to a final concentration of 0.25 mg ml^{-1} and refolded overnight in 0.9 M guanidine HCl, 0.1 M Tris pH 8.0, 1 mM L-cystine and 2 mM L-cysteine under a nitrogen atmosphere. Refolded trypsinogens were purified on a home-made ecotin affinity column, using a gradient from 50 mM Tris pH 8.0, 0.2 M NaCl to 50 mM HCl. Trypsinogens were activated by proteolytic cleavage with bovine enteropeptidase (Roche) in 100 mM Tris pH 8.0 and 1 mM $CaCl_2$, at a trypsinogen concentration of 0.1–0.2 mg ml^{-1}. For mesotrypsinogen and mesotrypsinogen-S195A, activations were conducted at a 1:300 (w/w) ratio of enteropeptidase to mesotrypsinogen for 3–4 h at 37 °C. For cationic trypsinogen and trypsinogen-S195A, activations were conducted at a 1:1000 (w/w) ratio of enteropeptidase to trypsinogen for 1 h at 37 °C. Anionic trypsinogen was autoactivated on ice for 2–3 h in the absence of enteropeptidase. Anionic trypsinogen-S195A activation was conducted at a 1:300 (w/w) ratio of enteropeptidase to trypsinogen for 3–4 h at 37 °C. Activated trypsins were purified on HiTrap Benzamidine FF affinity columns (GE Healthcare) by elution with a linear gradient from buffer 50 mM Tris-pH 8.0, 0.5 M NaCl to 30 mM HCl.

Competitive inhibition studies. The inhibition constant (K_i) of the selected APPI-3M variant toward each of the four tested serine proteases was determined by modifying the inhibition assay described in Cohen et al.[1], using a Synergy2 microplate 30 spectrophotometer (BioTek, Winooski, VT) at 37 °C. Specifically, the following conditions were employed in the experiments with mesotrypsin, cationic trypsin, and anionic trypsin: the substrate was Z-GPR-pNA in a fixed concentration of 150 µM; the inhibitor concentrations ranged between 100 pm and 550 pM; the enzyme concentration was 25 pM; the stock solution of each component was 40× the final concentration; the dilution buffers were an inhibitor dilution buffer (10 mM Tris, pH = 8.0, 0.1 mg ml^{-1} BSA), an enzyme dilution buffer (20 mM NaAc, pH = 4.5, 1 mg ml^{-1} BSA, 10 mM $CaCl_2$), and a binding buffer (100 mM Tris, pH = 8.0, 1 mM CaCl2, 1% BSA). The reaction was initiated after incubation of the components for 10 min at 37 °C, and the enzyme (8 µl) was added into a microplate (non-binding, 96 well microplate; Greiner, Kremsmuster, Austria) containing a mixture of substrate (8 µl), inhibitor (8 µl), and binding buffer (288 µl). The reaction with mesotrypsin was monitored for 1.5 h and the reactions with cationic and anionic trypsin were monitored for 4 h to achieve a steady-state rate. In the experiments with KLK6, the following conditions were employed: the substrate was BOC-FSR-MSC in a fixed concentration of 1 mM; the concentrations of the inhibitor ranged between 5 nM and 50 nM; the concentration of the enzyme was 1 nM; the stock solution of each component was 3× the final concentration; and the dilution buffer was a KLK6 assay buffer (50 mM Tris, pH = 7.3, 100 mM NaCl, 0.2% BSA). The reaction was initiated after incubating the components for 10 min at 37 °C, at which time the enzyme (100 µl) was added into a microplate (non-binding, 96-well microplate; Greiner) containing a mixture of the substrate (100 µl) and the inhibitor (100 µl). The reaction was monitored for 6 h.

The following tight-binding equation was used to calculate the inhibition constant (K_i): $\frac{V_0-V_i}{V_i}=\frac{[I_0]}{K_i(1+\frac{[S_0]}{K_m})}$, where Vi is the steady-state rate in the presence of the inhibitor, V_0 is the steady-state rate in the absence of the inhibitor, K_m is the Michaelis constant for substrate cleavage, and [S_0] and [I_0] are the initial concentrations of the substrate and inhibitor, respectively. Calculations were performed by using K_m values of 24.66 ± 1.30 µM for mesotrypsin, 22.84 ± 1.90 µM for cationic trypsin, 10.69 ± 0.65 µM for anionic trypsin, and 329.30 ± 2.50 µM for KLK6[1].

Protein crystallization and structure determination. Purified mesotrypsin was mixed with APPI-3M-T11V/G17R in a 1:5 molar ratio and subjected to crystallization trials using the sitting drop vapor diffusion method. Screening for the initial crystallization conditions was performed using the Index screening kit of Hampton Research at 293 K. Each drop contained a mixture of 0.3 μl of crystallization solution and 0.3 μl of the mesotrypsin_APPI-3M-T11V/G17R complex. Crystals grew after 48 h in a drop containing 0.1 M NaCl, 0.1 M Bis-Tris, pH 6.8, and 1.42 M ammonium sulfate. The crystals were harvested and flash-cooled in liquid nitrogen prior to data collection. X-ray diffraction data were collected at 100K at beamline ID30B of the European Synchrotron Radiation Facility (ESRF, Grenoble, France) to a maximum resolution of 2.3 Å. The crystals belong to P31 space group, with unit cell dimensions of a 53.215, b 53.215, and c 237.163, and they contain two molecules of mesotrypsin and two molecules of APPI-3M-T11V/G17R in the asymmetric unit.

The X-ray data of mesotrypsin – APPI-3M-T11V/G17R crystals was processed, merged, and scaled using XDS[4]. Data collection statistics are shown in Supplementary Table 3. Phase acquisitions and structure determination were performed by molecular replacement using Phaser[5] from the CCP4 Program Suite[6]. Protein Data Bank (PDB) code 5C67 [http://dx.doi.org/10.2210/pdb5C67/pdb] was used as the search model. Refinement was performed using Phenix.refine[7] and alternating rounds of model building and manual corrections were performed by COOT[8]. The coordinates and structure factors have been submitted to the Protein Data Bank under the accession codes 6GFI.

Supplementary Figures

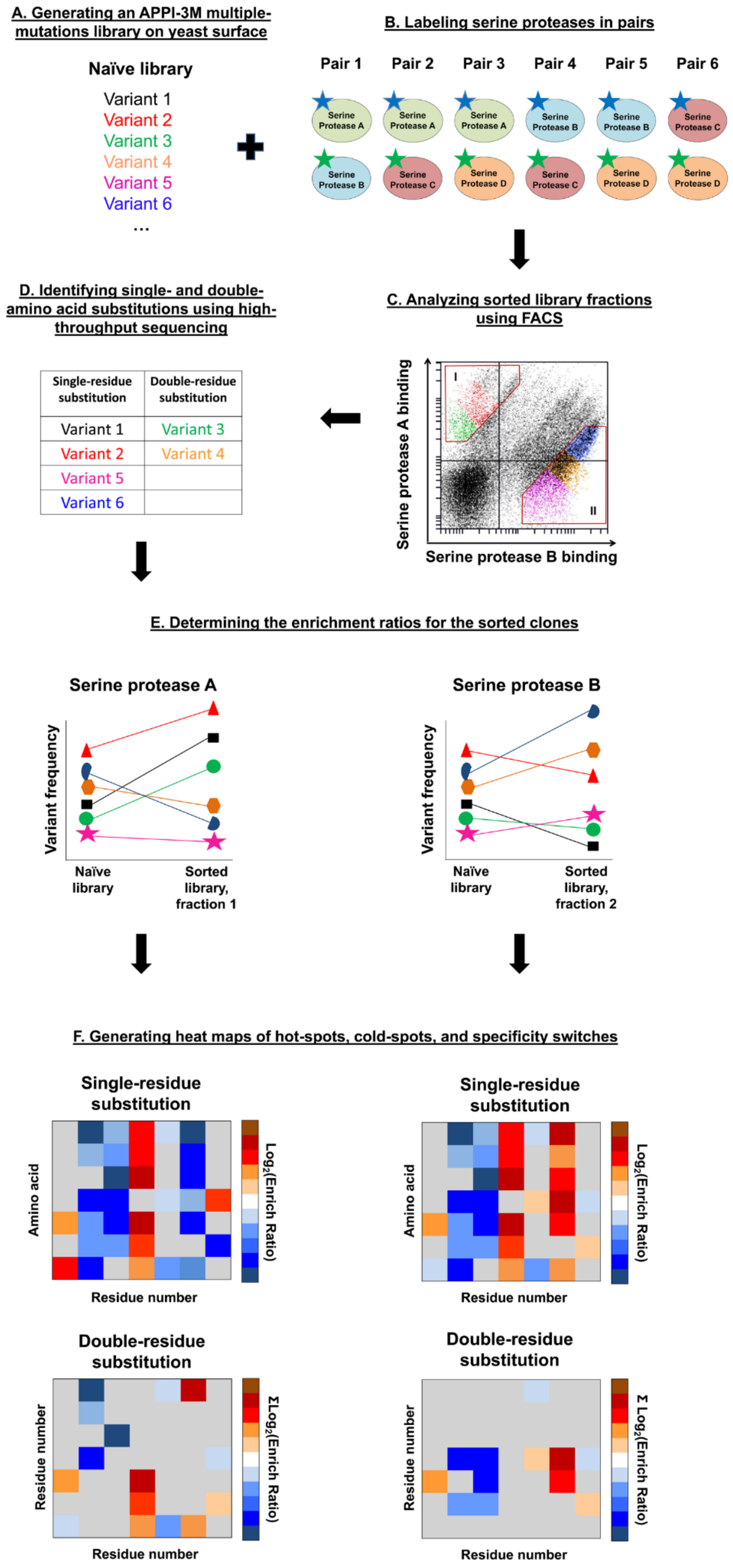

Supplementary Figure 1. Mapping the binding selectivity landscape. Illustration of the strategy used to map the binding selectivity landscape of APPI-3M to each of the four human serine proteases. We begin by generating a naïve APPI-3M library comprising random single- and double-residue mutations, displayed on the surface of yeast (**A**). Then, we divide the four human serine proteases to pairs combinatorially (six combinations altogether) and label each protease in the pair with a different fluorophore (**B**). After incubating the naïve APPI-3M library with each pair, we use fluorescence-activated cell sorting (FACS) to determine the optimal protease concentrations required to achieve maximal scattering of the binding signal between the different target pairs. Then, from each sort, we collect the two fractions of APPI-3M variants that bind exclusively to each of the proteases in the pair (**C**). Next, we use next-generation sequencing (NGS) to sequence these fractions (**D**) and analyze them computationally. Finally, using the calculated enrichment ratio of each variant (ER = frequency of a certain protease in the sorted library divided by its frequency in the naïve library) (**E**), we generate heat maps representing the binding selectivity (**F**).

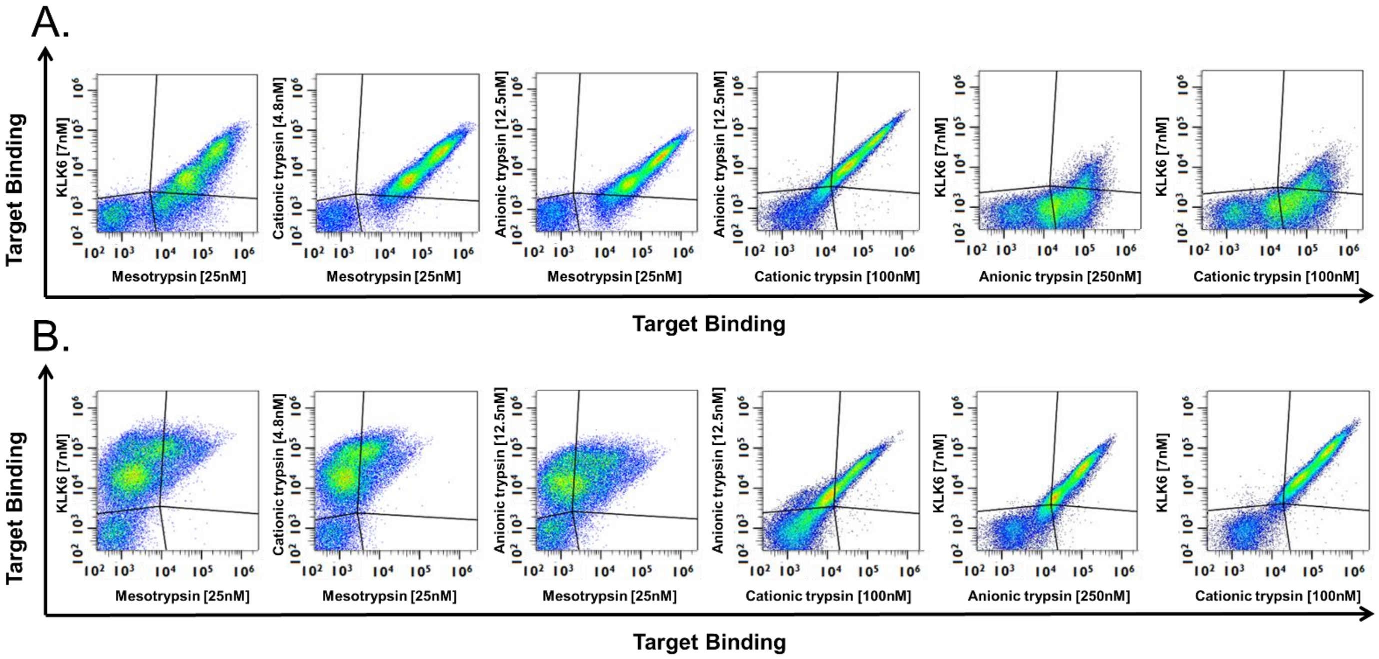

Supplementary Figure 2. Flow cytometry analysis of the sorted library. The selectivity of the variant populations that were collected from each sort was tested. (A) Populations of variants that were found to be highly selective toward the serine protease shown on the X axis (versus all others). (B) Populations of variants that were found to be highly selective toward the serine protease shown on the Y axis (versus all others). Green and blue indicate high and low cell densities, respectively.

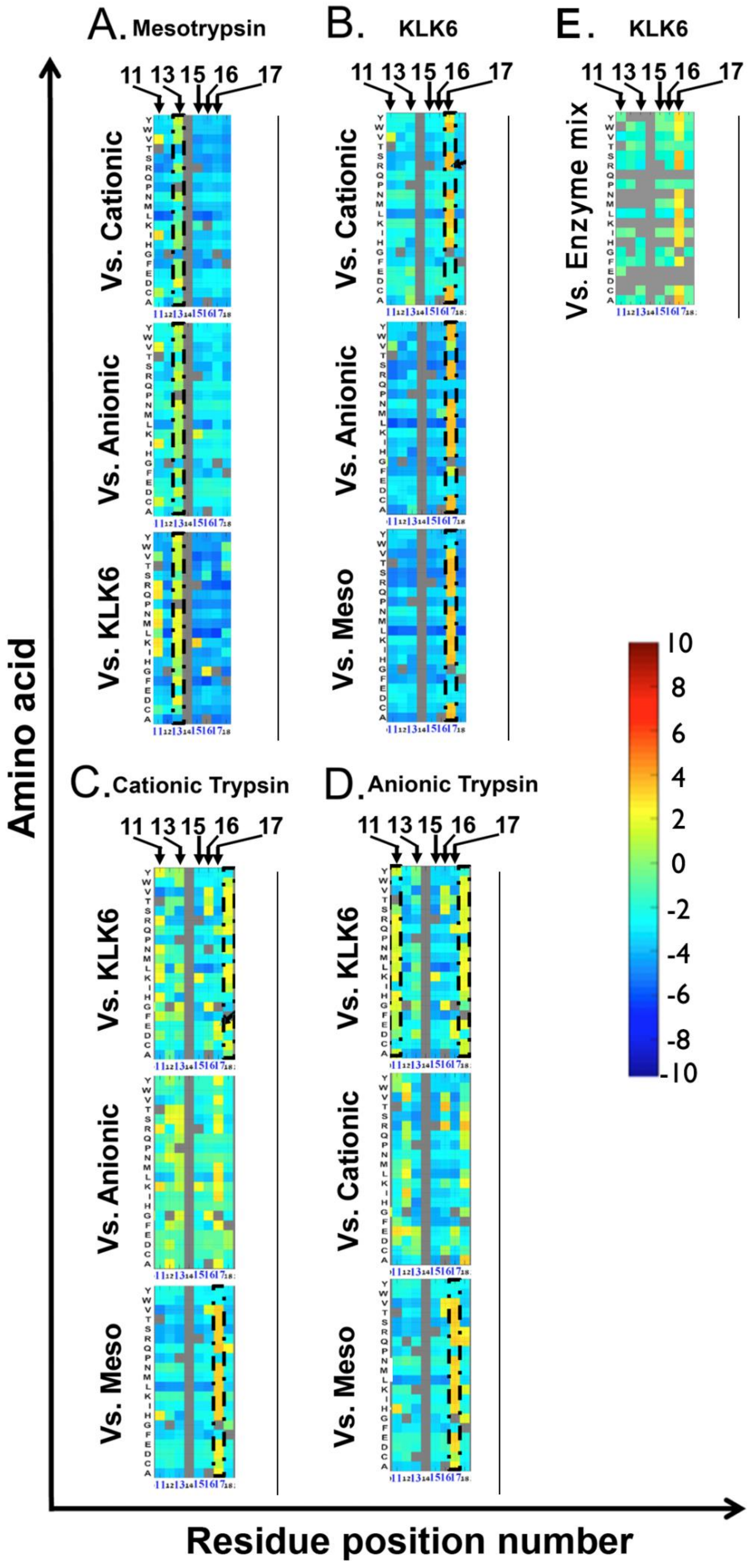

A. Mesotrypsin
B. KLK6
E. KLK6
C. Cationic Trypsin
D. Anionic Trypsin
Vs. Cationic
Vs. Anionic
Vs. KLK6
Vs. Meso
Vs. Enzyme mix
Amino acid
Residue position number

Supplementary Figure 3. APPI-3M binding loop selectivity landscape heat maps (enlargement of Fig. 2 A-D). The colors indicate the enrichment ratio and represent the effect of a single-amino acid substitution on the selectivity of APPI-3M toward one serine protease (A: mesotrypsin, B: KLK6, C: cationic trypsin, and D: anionic trypsin) versus each of the other three proteases. (E) Enrichment ratios heat map of APPI binding loop toward KLK6 versus a mixture of the other three proteases. The different colors in the heat maps correspond to the scale shown on the right panel and indicate log2 of the enrichment ratio [yellow and red: positive (increased selectivity); green: negative (decreased selectivity)]. The position of the substituted amino acid is shown on the X axis and the substituting amino acid is shown on the Y axis. Meso: mesotrypsin; Cationic: cationic trypsin; Anionic: anionic trypsin.

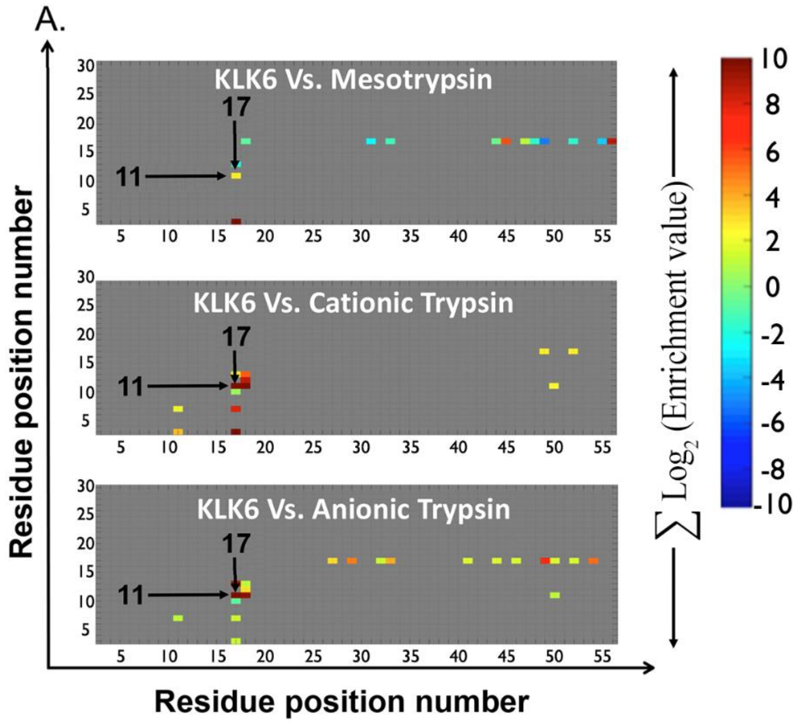

Supplementary Figure 4. Selectivity landscape of APPI-3M (enlargement of Fig. 3A)**.** The heat maps demonstrate the effect of double-amino acid mutations in APPI-3M on the selectivity toward KLK6 versus the three other serine proteases. The effect of different pairs of mutated residues on selectivity is illustrated by the colors of the heat maps [red = increased selectivity, enrichment ratio > 1; blue = decreased selectivity, enrichment ratio < 1]. The contribution of each double mutation to selectivity was summed and the maps demonstrate the overall effect. The X and Y axes indicate the position of the substituted amino acid residues.

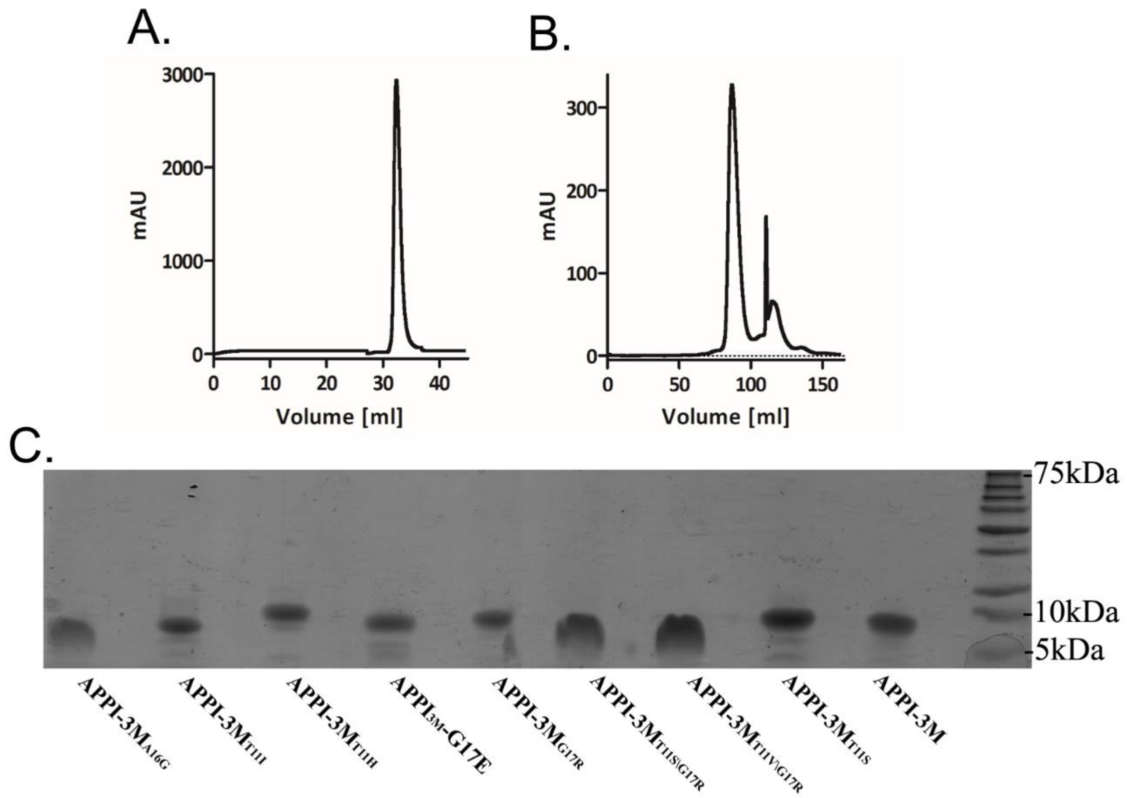

Supplementary Figure 5. Purification of APPI variants. An example of APPI-3M$_{T11V/G17R}$ purification using nickel/IMAC chromatography (A) and size-exclusion chromatography (B). The left peak in (B) is of APPI-3M$_{T11V/G17R}$, while the right peak is of a small molecule, presumably imidazole, which was used for elution in the nickel column. The same purification procedure was repeated for all selected variants. (C) SDS-PAGE of the purified APPI variants.

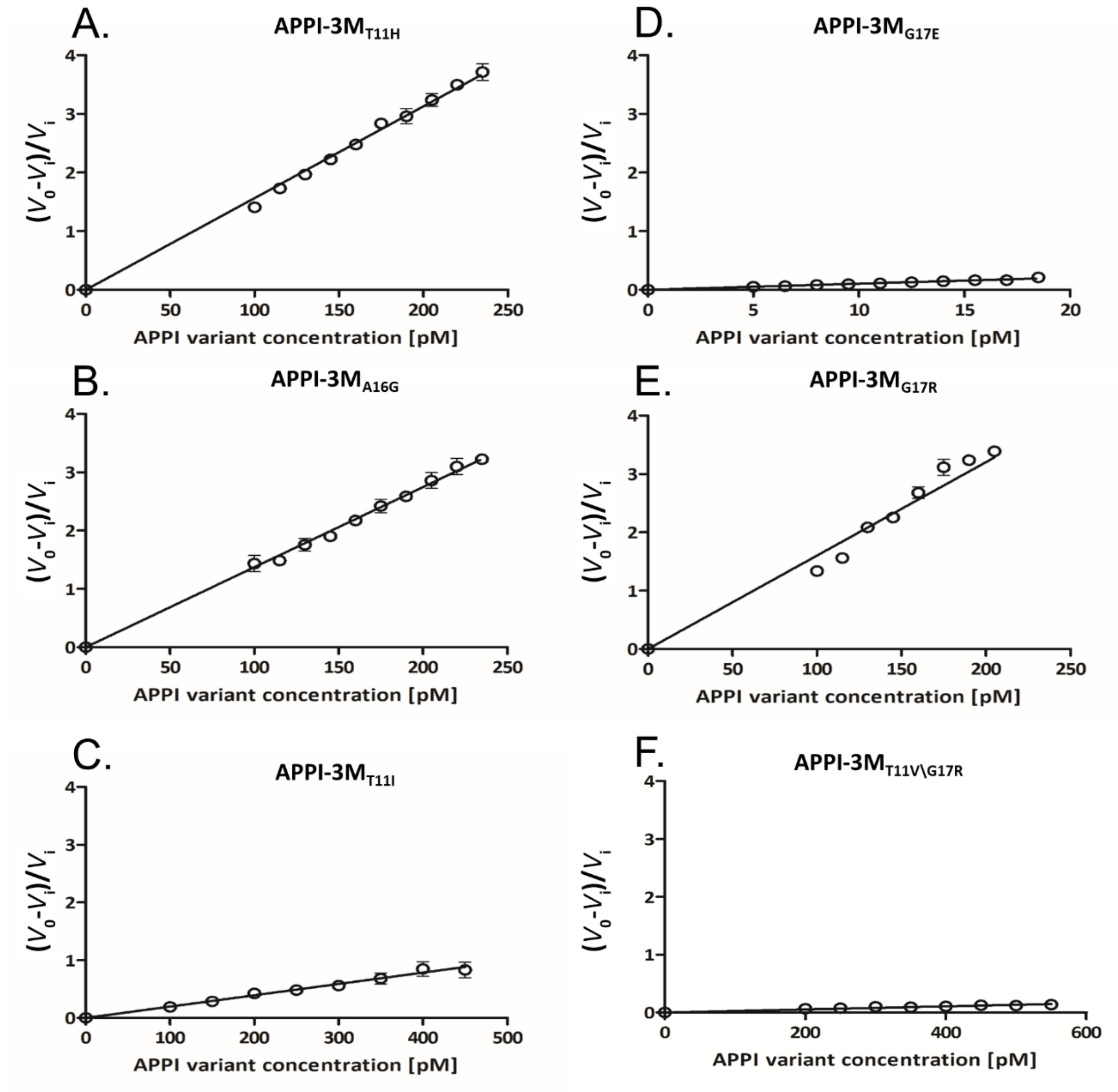

Supplementary Figure 6. Slow tight binding inhibition. Slow tight binding inhibition assay for mesotrypsin, anionic trypsin, cationic trypsin, and KLK6 by APPI-3M variants. The slope in each graph was used to calculate the K_i for (A) the inhibition of anionic trypsin by APPI-3M$_{T11H}$, (B) the inhibition of cationic trypsin by APPI-3M $_{A16G}$, (C) the inhibition of mesotrypsin by APPI-3M $_{T11I}$, (D) the inhibition of KLK6 by APPI-3M$_{G17E}$, (E) the inhibition of cationic trypsin by APPI$_{G17R}$, and (F) the inhibition of mesotrypsin by APPI-3M$_{T11V/G17R}$. V_0 represents the reaction rate without inhibitor, while V_i represents the reaction rate in the presence of the respective APPI-3M variant.

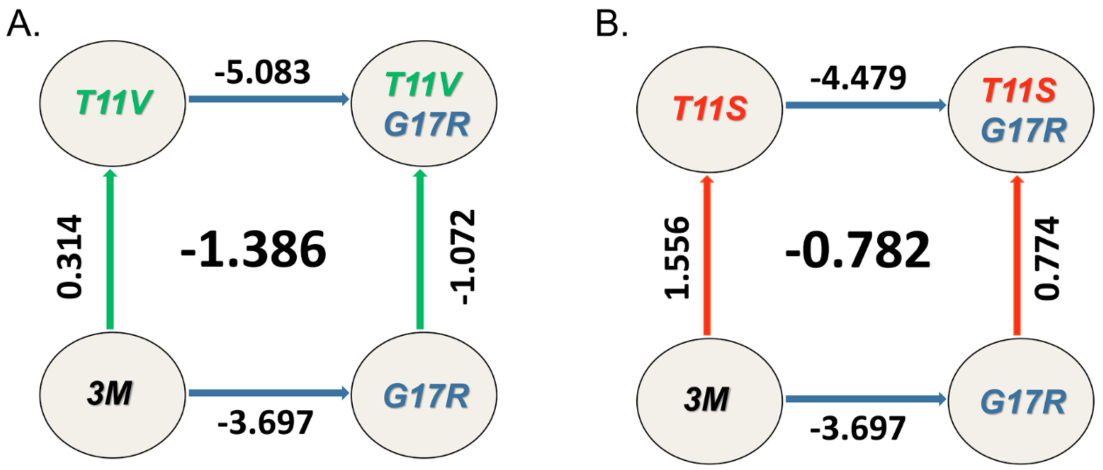

Supplementary Figure 7. Double-mutant cycle analysis. The cycle analysis is shown for measuring the coupling between residues 11 and 17 in KLK6 selectivity. (A) Free energy changes of the T11V/G17R substitution. (B) Free energy changes of the T11S/G17R substitution. To assess whether the effects of the mutations on the measured K_i are independent or correlated (cooperative), we assessed the strength of the interactions between two residues, *X* and *Y*, in the protein (*P*) in a cycle that comprised the wild-type protein P_{XY}, two single mutants, P_{X0} and P_{0Y}, and the corresponding double mutant, P_{00} (*0* indicates a mutation). A measure of the strength of the interaction between residues *X* and *Y* is considered the coupling energy, $\Delta\Delta G_{\text{int}}$, which is given by:

$$\Delta\Delta G_{\text{int}} = -RT\ln\left(\frac{S_{\text{XY}} \times S_{00}}{S_{0\text{Y}} \times S_{\text{X}0}}\right)$$

where *R* is the gas constant, *T* is the absolute temperature, and S_{X}, $S_{0\text{Y}}$, $S_{\text{X}0}$, and S_{00} correspond to the calculated total selectivity. A coupling energy of zero (i.e., additivity of mutational effects) indicates that *X* and *Y* do not interact. The free energy changes ($\Delta\Delta G$) upon a single point-mutation (i.e., the $\Delta\Delta G$ of P_{XY} and $P_{\text{X}0}$) were calculated in a similar manner, and are given by: $\Delta\Delta G = -RT\ln\frac{S_{\text{X}0}}{S_{\text{XY}}}$.

Each ellipse (corners) indicates a different APPI variant, as denoted, and the values near each arrow represent the $\Delta\Delta G$ (kcal mol^{-1}). The numbers at the middle of each panel indicate the coupling energy $\Delta\Delta G_{\text{int}}$ (kcal mol^{-1}).

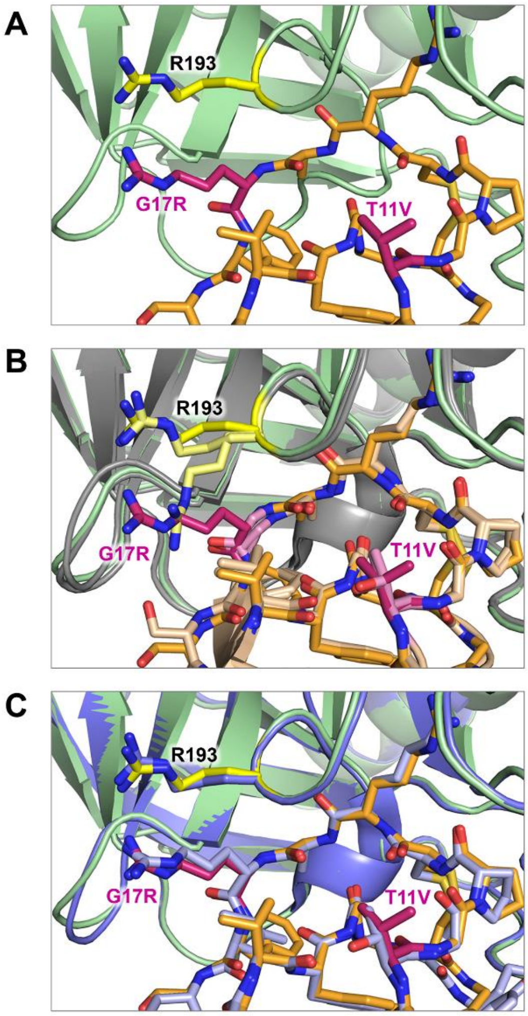

Supplementary Figure 8. Crystal structure of the mesotrypsin–APPI-3M-T11V/G17R complex. The structure reveals an unfavorable interaction between the mutant inhibitor and Arg-193 of mesotrypsin. (A) Arg-17 of the APPI-3M-T11V/G17R protein forms unfavorable steric and electrostatic interactions with Arg-193 of mesotrypsin. Mesotrypsin is shown in a *green cartoon representation*, with the Arg-193 side chain shown as *yellow sticks*; APPI-3M-T11V/G17R is shown as *orange sticks* with sites of mutations G17R and T11V shown in *dark pink*. (B) The conformation of Arg-193 of mesotrypsin is constrained by an interaction with Arg-17 of APPI-3M-T11V/G17R, as shown by superposition of the mesotrypsin/APPI-3M structure (mesotrypsin in *gray*, with Arg-193 as *pale yellow sticks*; APPI-3M as *pale orange and pale pink sticks*; PDB ID: 5C67 [http://dx.doi.org/10.2210/pdb5C67/pdb]). The two copies of mesotrypsin/APPI-3M present in the crystal lattice asymmetric unit reveal Arg-193 in two different conformations. The presence of Arg-17 in APPI-3M-T11V/G17R restricts Arg-193 of mesotrypsin to a single, more buried conformation. (C) The conformations of Arg-193 of Mesotrypsin and of Arg-17 of APPI-3M-T11V/G17R closely mirror the positions adopted by these residues in the complex of mesotrypsin with BPTI – a Kunitz domain inhibitor that only weakly inhibits mesotrypsin because of this disfavored interaction. The mesotrypsin/APPI-3M-T11V/G17R structure is superposed with the mesotrypsin/BPTI complex structure (PDB ID: 2R9P [http://dx.doi.org/10.2210/pdb2R9P/pdb]), shown with mesotrypsin in a *medium blue cartoon representation* and BPTI as *pale blue sticks*.

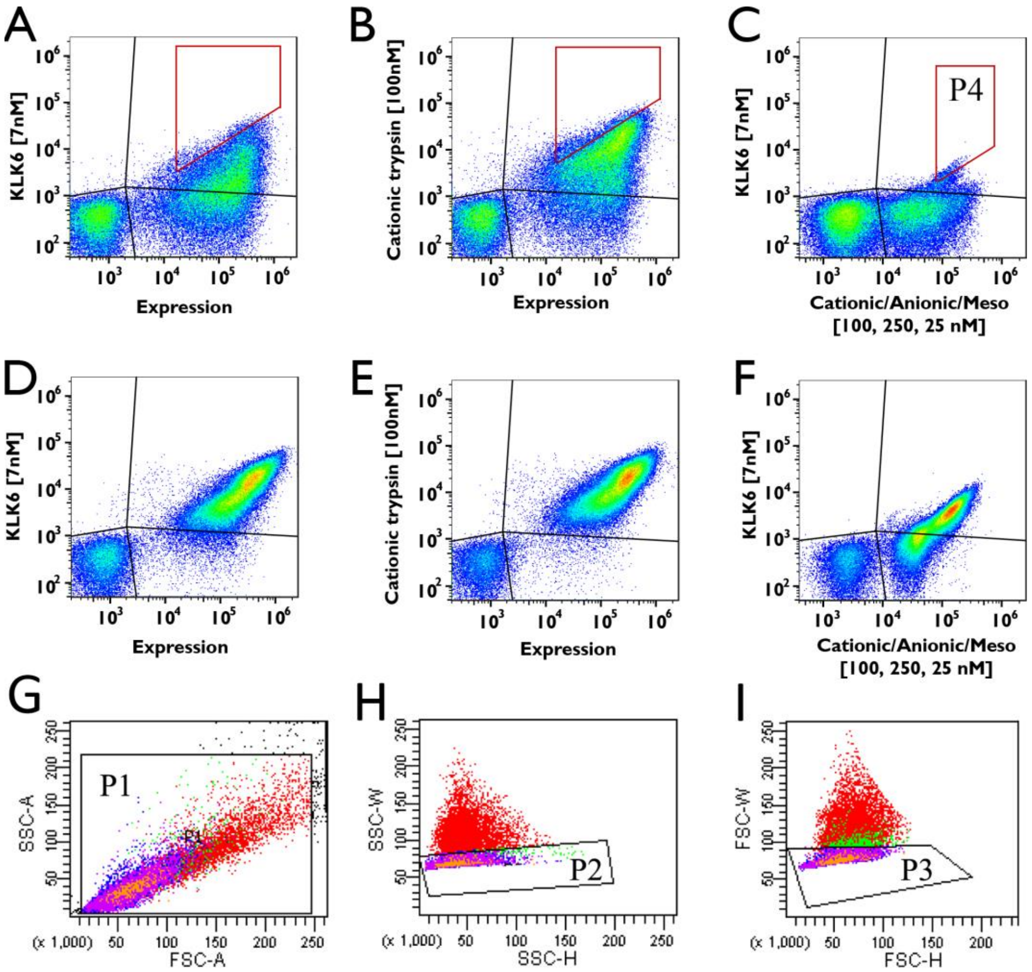

Supplementary Figure 9. Sequential affinity and multiple competitive screens. Sorting and flow cytometry analysis of sequential affinity and multiple competitive screens. (A, B) Flow cytometry sorting was used to screen the library to isolate APPI-3M variants with increased affinity toward KLK6 (A) or cationic trypsin (B). (C) Flow cytometry sorting was used to screen the library to isolate APPI-3M variants with increased selectivity toward KLK6 (Alexa Fluor-650) versus cationic trypsin, anionic trypsin and mesotrypsin (Alexa Fluor-488). In each sort (A, B and C), variant populations were collected inside the red gates. (D), (E), and (F) show flow cytometry analysis of the sorted populations from A, B and C respectively. Sequential affinity screens (A, B, D and E) were performed similar to the pairwise selectivity screen as described in the Methods, however, to detect the expression of the displayed proteins, we used a 9E10 mouse anti-c-Myc antibody (Abcam, Cambridge, UK, #cat ab32) in a 1:50 dilution, followed by a 1:50 dilution of phycoerythrin (PE)-conjugated anti-mouse secondary antibody (Sigma, Saint Louis, MO, #cat P9670). Multiple competition screen (C and F) were preformed similar to the pairwise selectivity screen, however for a mixture of four fluorescently labeled targets rather than for a pair. Green and blue indicate high and low cell densities, respectively. (G), (H) and (I) show the sorting protocol that used in the study (representative example that used for the sort from panel C). P1-P3 indicates the gates that used in the forward and side scatter (FSC and SSC) plots in which P1 is the first gate, P2 is the gate used for cells from P1 and P3 is the gate used for cells from P2. Finally, P4 (from panel C) is the gate used for cells from P3.

Supplementary Tables

Supplementary Table 1. Top 14 double mutants of APPI-3M with highest total selectivity

APPI-3M variant	Target	Selectivity versus competitor (calculated from enrichment ratio)				Total Selectivity[1]
		KLK6	Meso[2]	Cationic[2]	Anionic[2]	
T11V/G17R	KLK6	-	5.90E+04	3.64E+02	1.70E+02	3.65E+09
T11V/G17S	KLK6	-	1.65E+04	1.80E+02	6.54E+02	1.94E+09
T11P/G17S	KLK6	-	8.06E+04	1.02E+02	6.18E+01	5.10E+08
T11V/G17A	KLK6	-	3.99E+04	3.37E+02	2.78E+01	3.73E+08
T11V/G17L	KLK6	-	7.86E+02	4.52E+02	8.81E+02	3.13E+08
T11S/G17A	KLK6	-	4.21E+04	5.95E+01	1.00E+02	2.52E+08
T11L/G17R	KLK6	-	8.03E+04	7.91E+00	2.58E+02	1.64E+08
T11P/G17L	KLK6	-	7.02E+04	2.28E+01	6.00E+01	9.60E+07
T11S/G17R	KLK6	-	3.72E+04	2.37E+01	8.48E+01	7.49E+07
T11P/F18L	anionic	5.24E+02	2.40E+04	4.66E+00	-	5.84E+07
T11L/F18L	anionic	2.46E+02	2.24E+04	9.71E+00	-	5.36E+07
T11S/G17S	KLK6	-	3.64E+04	3.07E+01	4.38E+01	4.89E+07
T11R/G17S	cationic	4.80E+00	3.05E+04	-	2.52E+02	3.70E+07
T11G/F18L	anionic	1.91E+02	2.91E+04	4.52E+00	-	2.51E+07

[1]Total Selectivity is the multiplication of the three calculated selectivity values in each row (as marked in dashed line)

[2]Meso stands for mesotrypsin, Cationic stands for cationic trypsin, and Anionic stands for anionic trypsin

$$\text{Selectivity (NGS)} = \frac{\dfrac{ER_{mutant}\ \text{for Target}}{ER_{WT}\ \text{for Target}}}{\dfrac{ER_{mutant}\ \text{Competitor}}{ER_{WT}\ \text{Competitor}}}$$

Supplementary Table 2: The calculated selectivity of double-mutant APPI-3M variants toward KLK6 versus the three other proteases

Mutant	T11S/G17R	T11V/G17R
vs. mesotrypsin	37.21×10^3	58.97×10^3
vs. anionic trypsin	84.78	169.99
vs. cationic trypsin	23.74	364.04
KLK6 total selectivity	7.49×10^7	3.65×10^9

$$\text{Calculated Selectivity} = \frac{\dfrac{ER_{WT}\text{ for KLK6}}{ER_{mutant}\text{ for KLK6}}}{\dfrac{ER_{WT}\text{ for other protease in the pair}}{ER_{mutant}\text{ for other protease in the pair}}}$$

Supplementary Table 3: Data collection and refinement statistics (molecular replacement).

	**Mesotrypsin with APPI-3M-T11V/G17R**
**Data collection**	
Space group	P31
Cell dimensions	
a, *b*, *c* (Å)	53.215, 53.215, 237.163
α, β, γ (°)	90.0,90,0.,120.0
Resolution (Å)	46.09-2.30(2.38-2.30) *
R_{merge}	0. 11(0.93)
$I / \sigma I$	10.9(1.21)
Completeness (%)	99.7(98.2)
Redundancy	8.3(8.3)
**Refinement**	
Resolution (Å)	46.09-2.3
No. reflections	33, 190
R_{work} / R_{free}	24.54/30.12
No. atoms	4273
Protein	4257
Ligand/ion	4
Water	12
B-factors	81.95
Protein	81.98
Ligand/ion	79.11
Water	74.55
R.m.s. deviations	
Bond lengths (Å)	0.013
Bond angles (°)	1.28

*1 crystal was used for Meso_APPI_T11V_G17R structure determination
*Values in parentheses are for highest-resolution shell.

Supplementary Table 4: Comparison of enrichment values between sequential affinity and pairwise selectivity screens

Mutation	Sequential affinity Enrichment (KLK6)	[1,2]Pairwise selectivity Enrichment (KLK6+/[1]Cat-)	Sequential affinity Enrichment (Cationic)	[1,2]Pairwise selectivity Enrichment (Cat+KLK6-)
Unmodified	0	0.18	0	0.17
G17E	0	0.12	0	4.88
G17R	11.27	12.50	4.57	0.08

[1]Cat stands for Cationic trypsin
[2] '+' and '-' within protein pairs indicate from which sorting gate the enrichment values were taken. For example, (Protease A+/ Protease B-) indicates the gate that screens for protease A selectivity over protease B (the upper left gate in Supplementary Figure 1C).

Supplementary Table 5: The selectivity of APPI-3M$_{G17E}$ and APPI-3M$_{G17R}$ toward KLK6 and cationic trypsin

Mutation	[1]K_i for KLK6 (M)	Fold K_i for KLK6	[1]K_i for Cationic trypsin (M)	Fold K_i for Cationic	Selectivity (vs. APPI-3M)
Unmodified	$(3.62\pm0.1)\times10^{-10}$	1	$(2.25\pm0.06)\times10^{-11}$	1	1
G17E	$(4.64\pm0.39)\times10^{-9}$	0.078	$(8.58\pm0.59)\times10^{-11}$	0.26	3.4
G17R	$(7.74\pm0.26)\times10^{-11}$	4.6	$(8.12\pm0.08)\times10^{-12}$	2.7	1.7

[1]Results (means ±SD) were obtained from three independent experiments

Supplementary Table 6: Comparison of enrichment values between pairwise selectivity and multi-competition screens

	Multi-competition	Pairwise selectivity					
Mutation	[1,2]KLK6+ meso/cationic/ anionic(-)	[1,2]KLK6(+) Meso(-)	[1,2]KLK6(+) Cationic(-)	[1,2]KLK6(+) Anionic(-)	[1,2]KLK6(-) Meso (+)	[1,2]KLK6(-) Cationic (+)	[1,2]KLK6(-) Anionic(+)
G17R	17.99	12.29	12.50	13.30	0.08	0.08	0.02
T11S_G17R	4.16	7.38	17.84	16.54	0.73	0.45	0.04
T11V_G17R	3.04	5.11	24.72	13.48	0.07	0.18	0.02

[1] Meso stands for mesotrypsin, Cat stands for cationic trypsin, and Anionic stands for anionic trypsin
[2] '+' and '-' within protein pair indicate from which sorting gate the enrichment values were taken. For example, (Protease A+/ Protease B-) indicates the gate that screens for protease A selectivity over protease B (the upper left gate in Supplementary Figure 1C).

Supplementary Table 7: Comparison of calculated selectivity between NGS and enzymatic assays (K_i)

		Calculated selectivity[1,2] for KLK6					
	Variant	Vs. Mesotrypsin	Rank	Vs. Cationic	Rank	Vs. Anionic	Rank
From NGS	T11S	7.47	2	0.25	3	0.03	3
	T11V	0.24	3	**123.5**	2	44.84	2
	G17R	130360.87	1	153.91	1	480.51	1
From K_i	T11S	2.14	2	0.12	3	0.3	3
	T11V	0.64	3	**0.6**	2	1.58	2
	G17R	32.26	1	1.69	1	7.42	1
From NGS	T11S/G17R	37210	2	**23.74**	2	84.78	2
	T11V/G17R	58970	1	364.04	1	169.99	1
From K_i	T11S/G17R	31.62	2	**0.94**	2	3.87	2
	T11V/G17R	111.1	1	2.32	1	8.94	1

[1] Selectivity values greater than 1 indicate selectivity improvement toward KLK6

[2] Ranking is within each method and according to the level of improvement (the greatest improvement ranked as 1). See example for ranking comparison of mesotrypsin selectivity calculated using NGS (bolded box) and K_i (dashed bolded box).

$$\text{Calculated Selectivity (NGS)} = \frac{\dfrac{ER_{WT}\ \text{for KLK6}}{ER_{mutant}\ \text{for KLK6}}}{\dfrac{ER_{WT}\ \text{for other protease in the pair}}{ER_{mutant}\ \text{for other protease in the pair}}}$$

$$\text{Calculated Selectivity (Ki)} = \frac{\dfrac{Ki_{WT}\ \text{for KLK6}}{Ki_{mutant}\ \text{for KLK6}}}{\dfrac{Ki_{WT}\ \text{for other protease in the pair}}{Ki_{mutant}\ \text{for other protease in the pair}}}$$

REFERENCES

1 Cohen, I. *et al.* Combinatorial protein engineering of proteolytically resistant mesotrypsin inhibitors as candidates for cancer therapy. *Biochem J* **473**, 1329-1341, doi:10.1042/BJ20151410 (2016).

2 Laskowski Jr, M. & Sealock, R. W. in *The enzymes* Vol. 3 375-473 (Elsevier, 1971).

3 Salameh, M. A., Soares, A. S., Hockla, A. & Radisky, E. S. Structural basis for accelerated cleavage of bovine pancreatic trypsin inhibitor (BPTI) by human mesotrypsin. *J Biol Chem* **283**, 4115-4123, doi:10.1074/jbc.M708268200 (2008).

4 Kabsch, W. Xds. *Acta Crystallogr D Biol Crystallogr* **66**, 125-132, doi:10.1107/S0907444909047337 (2010).

5 McCoy, A. J. *et al.* Phaser crystallographic software. *J Appl Crystallogr* **40**, 658-674, doi:10.1107/S0021889807021206 (2007).

6 Winn, M. D. *et al.* Overview of the CCP4 suite and current developments. *Acta Crystallogr D Biol Crystallogr* **67**, 235-242, doi:10.1107/S0907444910045749 (2011).

7 Adams, P. D. *et al.* PHENIX: a comprehensive Python-based system for macromolecular structure solution. *Acta Crystallogr D Biol Crystallogr* **66**, 213-221, doi:10.1107/S0907444909052925 (2010).

8 Emsley, P. & Cowtan, K. Coot: model-building tools for molecular graphics. *Acta Crystallogr D Biol Crystallogr* **60**, 2126-2132, doi:10.1107/S0907444904019158 (2004).
